# Proton Transfer‐Driven Modification of 3D Hybrid Perovskites to Form Oriented 2D Ruddlesden–Popper Phases

**DOI:** 10.1002/smsc.202100114

**Published:** 2021-12-23

**Authors:** Zonghui Duan, Guangren Na, Shixun Wang, Jiajia Ning, Bangyu Xing, Fei Huang, Arsenii S. Portniagin, Stephen V. Kershaw, Lijun Zhang, Andrey L. Rogach

**Affiliations:** ^1^ Department of Materials Science and Engineering Centre for Functional Photonics (CFP) City University of Hong Kong Hong Kong SAR 999077 P. R. China; ^2^ State Key Laboratory of Superhard Materials Key Laboratory of Automobile Materials of MOE College of Materials Science and Engineering Jilin University Changchun 130012 P. R. China; ^3^ Institute for Advanced Materials and Technology University of Science and Technology Beijing Beijing 100083 P. R. China

**Keywords:** aliphatic alkylamines, green emission, hybrid halide perovskites, proton transfer, Ruddlesden–Popper perovskite

## Abstract

Herein, it is shown how a proton transfer process between the organic moiety in 3D methylammonium lead halide perovskite and the introduced aliphatic alkylamines provides the basis for a fabrication route toward hybrid 3D/2D perovskites and finally purely 2D Ruddlesden–Popper (RP) perovskite phases, predominantly the *n* = 1 phase. Five alkylamines with varying aliphatic chain lengths, such as butylamine, octylamine, dodecylamine, hexadecylamine, and octadecylamine as antisolvents in toluene, are used, which quickly protonate during the spin‐coating deposition of thin perovskite films. Formation of hydrogen bonds between protonated alkylamines and lead halide slabs leads to mixed 3D/2D hybrid perovskites, where the ratio between the 3D and 2D phases can be adjusted by the concentration of the alkylamine containing antisolvents. Longer‐chain aliphatic alkylamines (12 carbon atoms or greater) are most prone to slice 3D perovskite into layered perovskites with efficient green emission reaching up to 38% for their photoluminescence quantum yield in films. Above a certain concentration threshold, 3D perovskite can be completely modified into 2D RP perovskite phases with crystalline orientation parallel to the substrate. The introduced facile perovskite phase modification approach provides a convenient way toward different kinds of 2D RP metal halide perovskite films with attractive optical properties.

## Graphical abstract

A proton transfer process between the methylammonium cations in the 3D lead halide perovskite framework and the introduced alkylamine additives with varying aliphatic chain lengths results in the formation of composite 3D/2D hybrid perovskites with efficient green emission, and finally purely 2D Ruddlesden–Popper (RP) perovskite films with attractive optical properties.

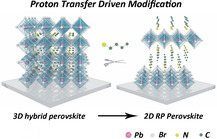

## 1 Introduction

Owing to outstanding optical and electronic properties, 3D organic–inorganic (also called “hybrid”) metal halide perovskites have recently been intensively investigated in the contexts of solar cells, light‐emitting devices, lasers, and photodetectors.^[^ ^1^ ^]^ However, their environmental instability severely hinders practical commercialization.^[^ ^2^ ^]^ In contrast, 2D Ruddlesden–Popper (RP) perovskites show promising stability due to the presence of long hydrophobic alkyl chain exteriors, which are able to protect perovskite slabs from moisture and inhibit the process of ion migration.^[^ ^3^ ^]^ The formula of 2D RP perovskites is (RNH_3_)_2_A_*n*‐1_M_*n*_X_3*n*+1_ (with *n* being an integer), where [RNH_3_]^−^ is an aliphatic or aromatic alkylammonium cation, A^+^ is an organic cation such as CH_3_NH_3_^+^ (methylammonium, abbreviated as MA) or HC(NH_2_)_2_^+^ (formamidinium, FA), M^2+^ is a metal cation such as Pb^2+^ or Sn^2+^, and X is a halide (Cl^−^, Br^−^, or I^−^) anion. In 2D RP perovskites, *n* layers of corner‐sharing or face‐sharing [MX_6_]^4−^ octahedral units with a large dielectric constant are sandwiched between low‐dielectric‐constant organic ammonium barrier layers, thus forming a quantum well‐like structure.^[^ ^4^ ^]^ The large dielectric constant contrast between the organic and inorganic components gives rise to extremely large exciton binding energy, which renders 2D RP perovskites useful optical properties, such as giant two‐photon absorption cross section^[^ ^5^ ^]^ and strong photoluminescence (PL).^[^ ^6^ ^]^

A variety of methods have been adopted for the fabrication of 2D RP perovskite films. In most instances, those methods can be grouped into three categories: one‐step deposition, two‐step deposition, and solution cation exchange. In a one‐step deposition process, 2D RP perovskite films are directly deposited from the precursor solutions which are prepared by dissolving 2D RP perovskite ingredients in *N*,*N*‐dimethylformamide (DMF) or dimethyl sulfoxide (DMSO) solvent.^[^ ^7^ ^]^ In a two‐step deposition variant, a PbI_2_ layer is first deposited after spin‐coating. Subsequently, the ammonium iodide dissolved in isopropanol is added onto the PbI_2_ layer, followed by a further spinning stage.^[^ ^8^ ^]^ The 2D RP perovskite film is then formed by intercalation of alkylammonium iodide into the PbI_2_ layer. In the third category, solution cation exchange can occur when alkylammonium halide salts pre‐dissolved in toluene are dripped onto a 3D perovskite film, or when a 3D perovskite film is soaked in alkylammonium halide solutions.^[^ ^9^ ^]^ All abovementioned methods to prepare 2D RP perovskite films involve ammonium halide salts. Zhao et al. introduced phenylalkylamines to FAPbI_3_ perovskite by a post‐deposition method.^[^ ^10^ ^]^ The defective regions at the perovskite film surfaces and grain boundaries were well passivated by these alkylamines, and the stability of the films was enhanced due to the hydrophobic aromatic group. However, phenylalkylamines anchoring on the surface of FAPbI_3_ perovskite films could not induce the formation of any new perovskite phases. On the contrary, when benzylamine was introduced to FA_0.15_Cs_0.85_Pb(I_0.73_B_0.27_)_3_, a thin 2D perovskite layer was found on the top of the 3D perovskite.^[^ ^11^ ^]^ Kim et al. added benzylamine to MAPbBr_3_ precursor solution and claimed that a proton transfer process between the MA cation and benzylamine enabled the formation of the 3D/2D hybrid perovskite combination.^[^ ^12^ ^]^ It is thus an important task to thoroughly study mechanisms of 3D/2D and 2D perovskite formation induced by alkylamines.

In this work, we directly dissolved aliphatic alkylamines in toluene serving as an antisolvent. The proton transfer between the methylammonium cations of 3D MAPbBr_3_ perovskite and alkylamines during the spin‐coating process allowed us to obtain both composite 3D/2D hybrid perovskites and highly oriented fully 2D RP perovskite films. The proportion of the resulting 2D RP perovskite within the hybrid 3D/2D film could be easily tuned by controlling the concentration of the alkylamine containing antisolvents. We demonstrated that the alkylamines with different chain lengths had significant effects on 2D RP perovskite formation. The alkylamines with longer chain length (12 and more carbon atoms) were more prone to transform the 3D perovskite frameworks into the layered 2D structures due to the lower formation energy.

## 2 Results and Discussion

**Figure 1:**
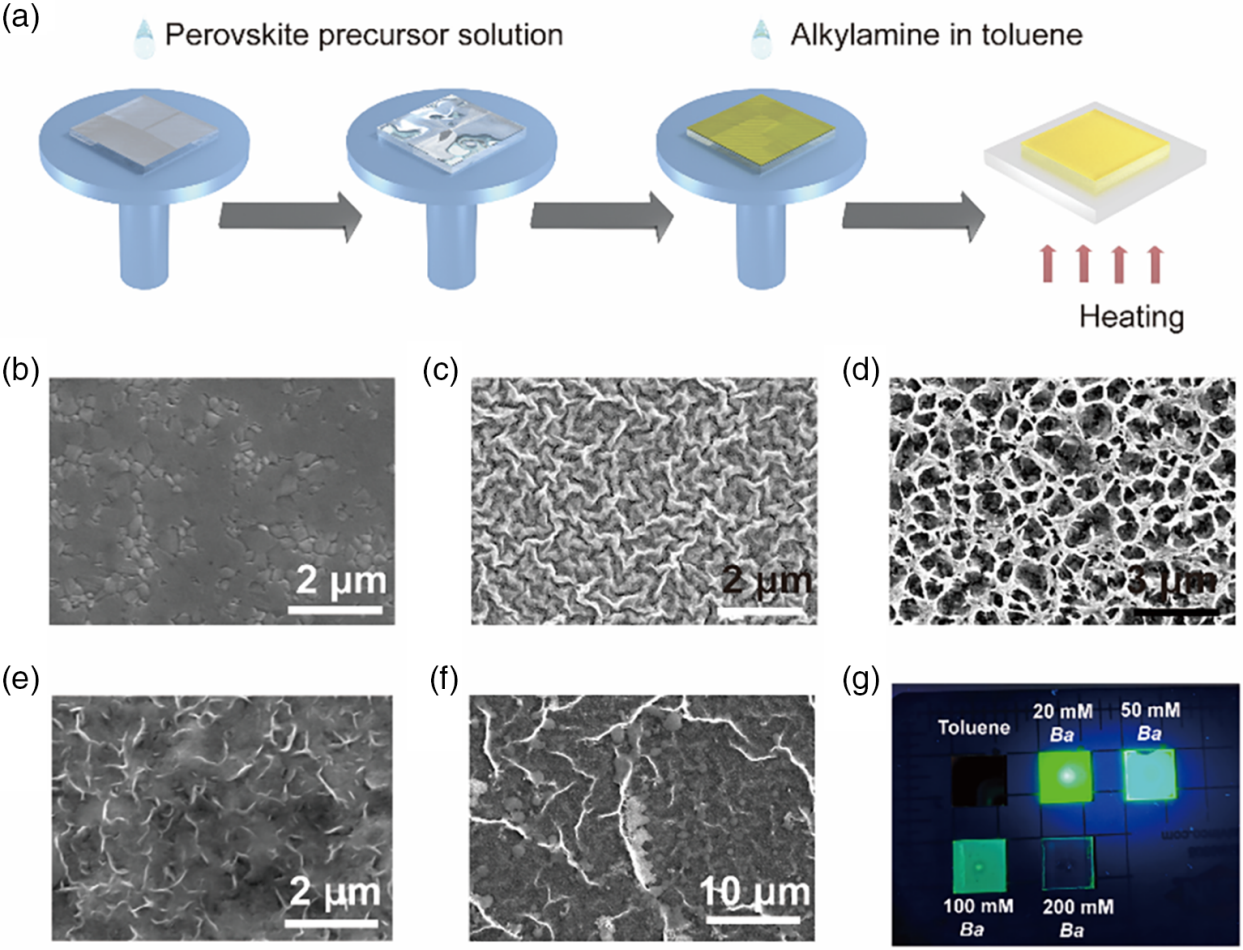
a) Scheme illustrating fabrication of perovskite films. SEM images of MAPbBr_3_ films fabricated using b) pure toluene, c) 20 mM *Ba*, d) 50 mM *Ba*, e) 100 mM *Ba*, and f) 200 mM *Ba*, respectively. g) Photograph demonstrating light emission of the MAPbBr_3_ film fabricated using pure toluene (no emission visible under UV lamp), and the films obtained with different amounts of *Ba*, as labelled on the photomontage (green emissions with varying intensities under UV lamp).

A schematic diagram of the fabrication of perovskite films is given in **Figure** **1**a. The perovskite films were fabricated by one‐step spin‐coating of the perovskite precursor (MAPbBr_3_ dissolved in DMSO) on glass substrates, followed by applying either pure toluene or a number of different alkylamines (or their different proportions) dissolved in toluene as antisolvents. A detailed description of these fabrication procedures is given in the Experimental Section. The antisolvents were introduced into the still spinning perovskite precursor films to promote the formation of the MAPbBr_3_ film, which would interact with the introducing long‐chain aliphatic alkylamine molecules through the dative bonds between nitrogen and lead ions, and at last, induce cation replacement through redox reaction to form layered RP perovskites.^[^ ^13^ ^]^ As shown in Figure 1b, the MAPbBr_3_ grains packed densely and uniformly when pure toluene was used as an antisolvent. Well‐facetted grains and grain boundaries are observed in the atomic force microscopy (AFM) image in Figure S1a, Supporting Information. When butylamine (abbreviated as *Ba*), a short‐chain aliphatic alkylamine containing four carbon atoms, was dissolved in toluene and introduced into the perovskite films, the film morphology drastically changed. From here on, we will denote the MAPbBr_3_ treated with 20, 50, 100, and 200 mM *Ba* in toluene as MAPbBr_3_/Ba20, MAPbBr_3_/Ba50, MAPbBr_3_/Ba100, and MAPbBr_3_/Ba200, respectively. The MAPbBr_3_/Ba20 film exhibited a dendritic texture as shown in Figure 1c and Figure S1b, Supporting Information. In the case of MAPbBr_3_/Ba50, the perovskite film became a honeycomb‐like structure with many micron‐sized holes on the surface of the film, which may be caused by the release of the methylamine gas during annealing (Figure 1d and Figure S1c, Supporting Information). In the case of MAPbBr_3_/Ba100, the perovskite grains were not observable anymore (Figure 1e); instead, the perovskite showed flake‐like morphology, which is also seen in the AFM image in Figure S1d, Supporting Information. In the case of MAPbBr_3_/Ba200, the film composed of large flakes became discontinuous, as shown in Figure 1f and Figure S1e, Supporting Information.

**Figure 2:**
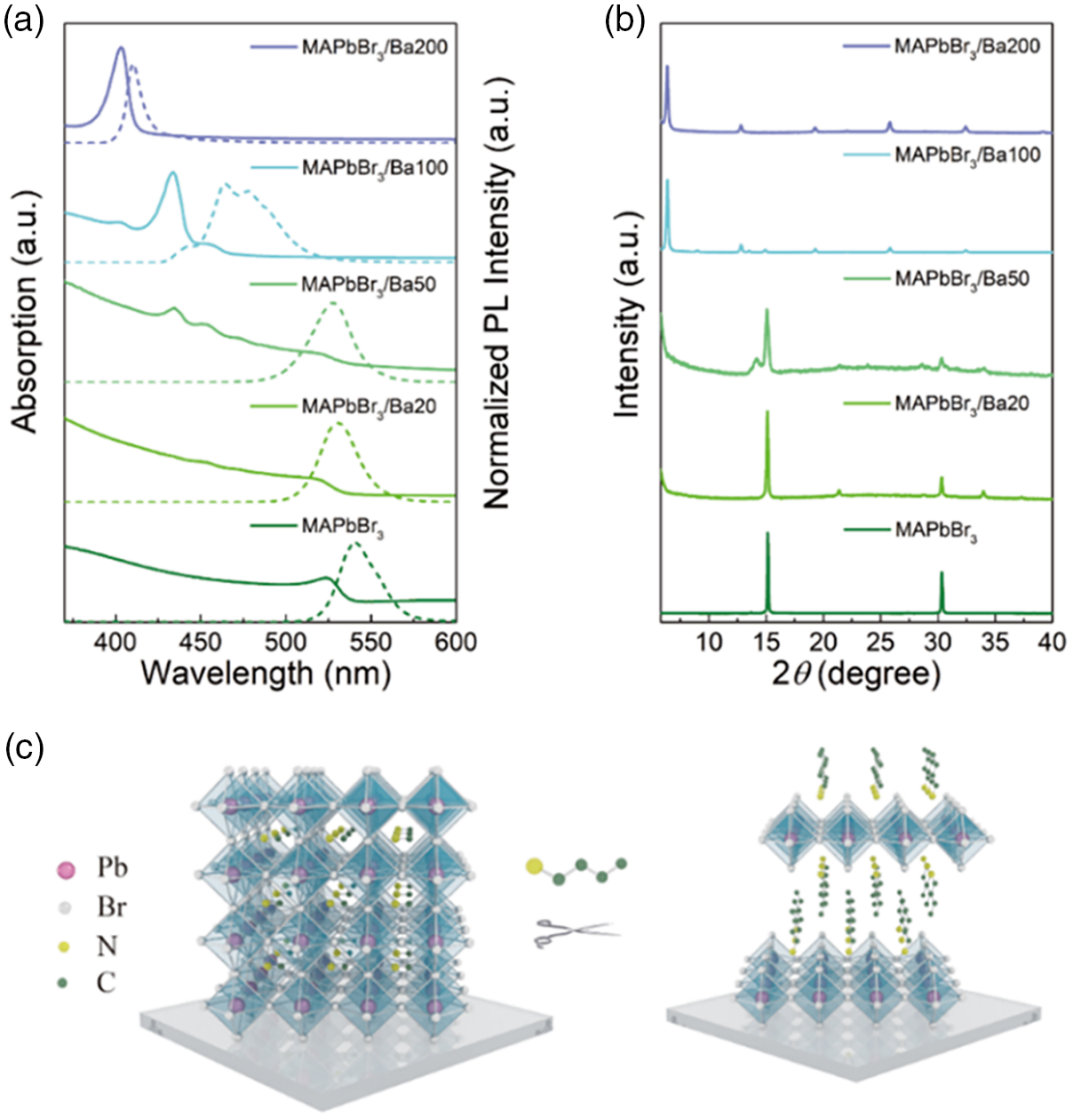
a) Absorption (solid lines) and PL (dashed lines) spectra of MAPbBr_3_ perovskite films fabricated using pure toluene, and 20 mM *Ba*, 50 mM *Ba*, 100 mM *Ba*, and 200 mM *Ba* solutions as antisolvents. b) XRD patterns of the perovskite films shown in (a). c) Schematic diagram showing the transformation of bulk MAPbBr_3_ perovskite under the action of butylamine (*Ba*).

In contrast to the MAPbBr_3_ film which showed no emission under a UV lamp, strong green PL could be easily observed for MAPbBr_3_/Ba20, MAPbBr_3_/Ba50, and MAPbBr_3_/Ba100 films under 365 nm excitation (Figure 1g). The brighter emission in the center of perovskite film may arise from the uneven distribution (concentration/degree of contact) of amines exposed at the film surface that promoted the level of transformation from 3D to 2D to a different degree. As the MAPbBr_3_ was treated with 200 mM *Ba* antisolvent, the margins of the obtained film showed cyan emission while the center of the film showed no visible PL. Under the room light, the color of the films also varied with *Ba* concentration (Figure S1f, Supporting Information): as an example, the color of the pristine MAPbBr_3_ film was orange, while the MAPbBr_3_/Ba200 film was almost transparent. The almost imperceptible emission and near‐transparent film appearance suggest an enlarged bandgap of the prepared perovskite film, which has little absorption in the visible range. Absorption and PL spectra of the MAPbBr_3_ films treated with different concentrations of *Ba* show a further remarkable difference in their optical properties (**Figure** **2**a). With increasing concentrations of *Ba* in toluene, a series of excitonic peaks in the absorption spectra appeared at shorter wavelengths. By referring to previously published data,^[^ ^14^ ^]^ the excitonic absorption peaks at 403, 434, 453, and 472 nm can be assigned to *n* = 1, *n* = 2, *n* = 3, and *n* = 4 phases of (BA)_2_MA_*n*‐1_Pb_n_Br_3*n*+1_ perovskite, respectively (the abbreviation “BA” here and later on stands for butylammonium). The multiple excitonic peaks indicate the mixture of phases present in MAPbBr_3_/Ba50 and MAPbBr_3_/Ba100 samples. Although the film of MAPbBr_3_ treated with 100 mM *Ba* is predominantly composed of the *n* = 2 phase, its emission intensity is much weaker than the high order (*n* > 2) phases. The dominant emission peak from *n* > 2 phases in the PL spectrum can be ascribed to the efficient energy cascade from low order layered perovskite to high order layered perovskite.^[^ ^15^ ^]^ Thus, the hybrid 2D/3D perovskite could exhibit efficient green emission (Figure 1g). The MAPbBr_3_/Ba200 film showed a dominant and strong excitonic peak at 403 nm and a symmetric PL peak at 410 nm, which indicates the formation of (BA)_2_PbBr_4_ (*n* = 1). In general, *n* = 1 layered perovskites possess an emission tail at a longer wavelength because of the easy formation of self‐trapped excitons.[^7^, ^16^] The symmetrical PL with a small full width at half maximum (FWHM) of just 12 nm indicates high quality film of (BA)_2_PbBr_4_.

Phase structures of the films were characterized by X‐ray diffraction (XRD). As shown in Figure 2b, the diffraction peaks located at 15° and 30.4° suggest the presence and preponderance of the cubic phase of MAPbBr_3_ perovskite for the sample made with pure toluene. When *Ba* was introduced, several additional diffraction peaks appeared, indicating the formation of other perovskite phases. For the MAPbBr_3_/Ba100 film, (100) and (200) planes corresponding to bulk MAPbBr_3_ perovskite indices almost vanished, and a strong XRD peak centered at 6.5° appeared, which can be indexed to the (002) plane of (BA)_2_PbBr_4_ perovskite.^[^ ^17^ ^]^ For the MAPbBr_3_/Ba200, XRD peaks corresponding to the MAPbBr_3_ perovskite disappeared totally, giving way to periodic diffraction peaks which correspond to the (002), (004), (006), (008), and (0010) planes of *n* = 1 (BA)_2_MA_*n*‐1_Pb_n_Br_3*n*+1_ 2D RP perovskite. Strong reflections from these (00*l*) planes indicate that inorganic perovskite slabs were grown mostly parallel to the substrates.^[^ ^18^ ^]^

We also prepared MAPbI_3_ perovskites using similar treatments with different amounts of *Ba* in toluene, ranging from 50 to 300 mM *Ba*. As shown in Figure S2, Supporting Information, both absorption and PL spectra of these films indicate evidence of large changes. With the increased concentration, the absorption band edges and PL peaks blueshift to shorter wavelengths. The excitonic peaks at 512, 570, 608, and 644 nm are assigned to the *n* = 1, *n* = 2, *n* = 3, and *n* = 4 phases of (BA)_2_MA_*n*‐1_Pb_n_I_3*n*+1_ perovskites.^[^ ^19^ ^]^ Similar to MAPbBr_3_ treated with *Ba* antisolvents, the MAPbI_3_/Ba50 and MAPbI_3_/Ba100 films have mixed phases of (BA)_2_MA_*n*‐1_Pb_n_I_3*n*+1_ indicated by the multiple excitonic peaks in their absorption spectra (Figure S2, Supporting Information). In the case where 300 mM *Ba* was used, the MAPbI_3_/Ba300 film showed a strong excitonic peak at 512 nm and a narrow PL peak at 522 nm with an FWHM of 19 nm. Therefore, the MAPbI_3_/Ba300 film was composed of a pure (BA)_2_PbI_4_ phase.

**Figure 3:**
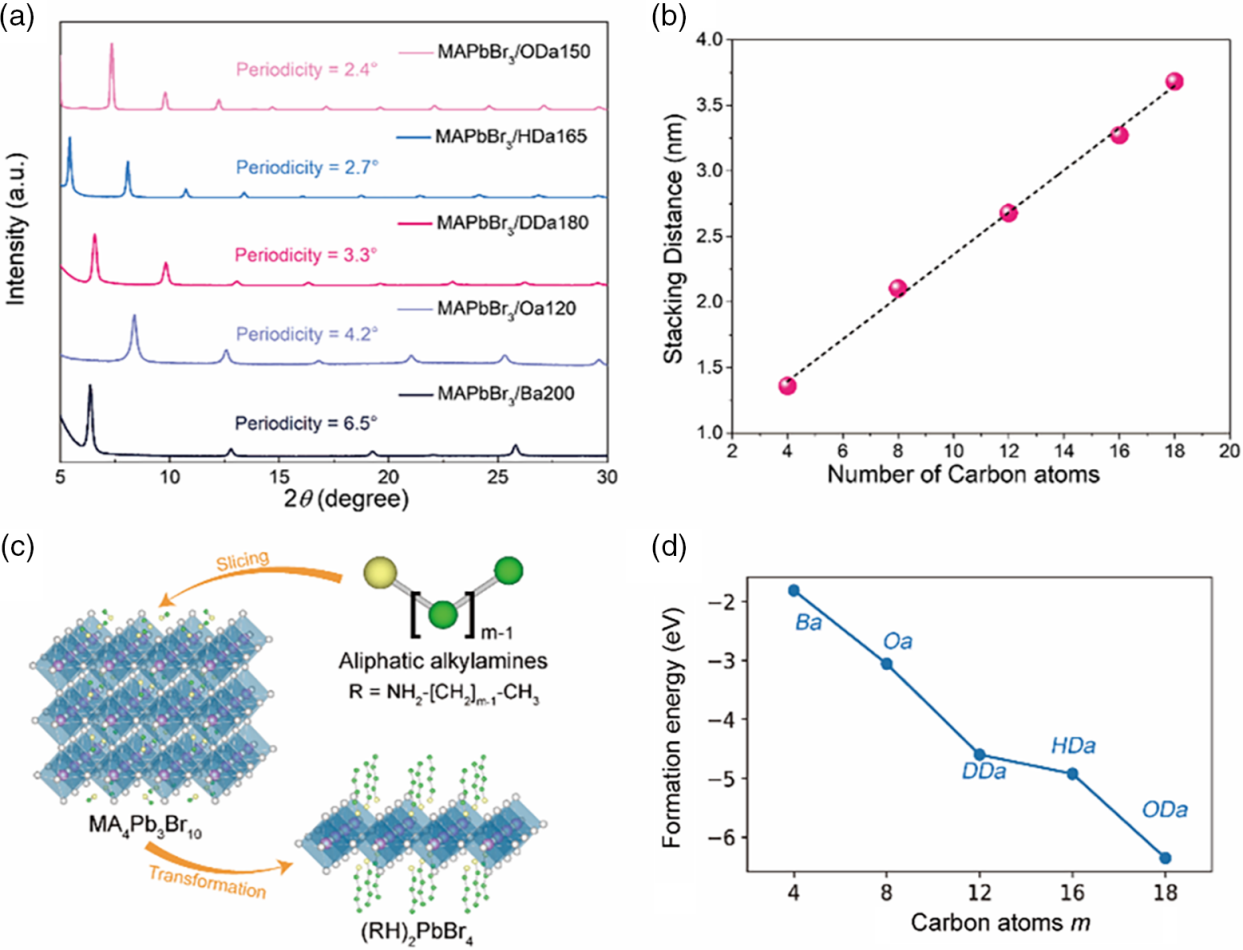
a) XRD patterns of different *n* = 1 phase layered perovskite films, as indicated on the frame. b) Relationship between the stacking distance of perovskite slabs determined from the Bragg relationship and the number of carbon atoms in the alkylamines used. c) Schematic diagram of aliphatic alkylamines R with varying lengths of the carbon chains slicing an *n* = 3 phase (MA)_4_Pb_3_Br_10_ perovskite into the *n* = 1 phase (RH)_2_PbBr_4_ structure. d) Calculated formation energies of *n* = 1 (RH)_2_PbBr_4_, with aliphatic alkylamines R = *Ba* (number of carbon atoms *m* = 4), *Oa* (*m* = 8), *DDa* (*m* = 12), *HDa* (*m* = 16), and *ODa* (*m* = 18).

Apart from using a short‐chain butylamine with four carbon atoms, we further explored the effects of different aliphatic alkylamines, systematically varying the lengths of the carbon chains, namely octylamine (*Oa*, 8 carbon atoms), dodecylamine (*DDa*, 12 carbon atoms), hexadecylamine (*HDa*, 16 carbon atoms), and octadecylamine (*ODa*, 18 carbon atoms) on the MAPbBr_3_. As can be judged from the optical spectra and XRD patterns of those samples, which are shown in Figures S3 and S4, Supporting Information, all these alkylamines dissolved in toluene could transform the 3D MAPbBr_3_ perovskite into layered structures (Figure 2c). Strikingly, when *ODa* was utilized, only the *n* = 1 layered perovskite could be yielded, which showed two distinct emission peaks in the 3D/2D hybrid perovskite films (Figure S4, Supporting Information). XRD patterns of MAPbBr_3_ films fabricated using 200 mM *Ba*, 120 mM *Oa*, 180 mM *DDa* 40, 165 mM *HDa*, and 150 mM *ODa* are provided in **Figure** **3**a, and their absorption and PL spectra are shown in Figure S5, Supporting Information. Although the XRD patterns and absorption spectra of the MAPbBr_3_/Oa120 film indicate that it is mostly composed of *n* = 1 phase 2D perovskite, a weak PL peak located at 430 nm still can be observed, which can be ascribed to the presence of a minor amount of *n* = 2 phase 2D perovskite; at the same time, an efficient energy funneling from the *n* = 1 phase to the *n* = 2 phase is taking place. The periodicity of the XRD peaks varied according to the length of the alkylamine used for film fabrication, indicating the formation of the *n* = 1 phase of 2D RP perovskites (Figure 3a). From the 2*θ* values, the stacking distance between perovskite inorganic slabs was calculated, and is summarized in Figure 3b. The linear relationship between the stacking distance (as derived from the Bragg relationship) and the number of carbon atoms in the alkylamines is perfectly consistent with the increased chain length. Billing and Lemmerer thoroughly studied the crystal structure and phase transitions of (C_*m*_H_2*m*+1_NH_3_)_2_PbI_4_ (*m* = 4–10, 12, 14, 16, and 18) perovskites and found that the interlayer spacing increased with the length of the alkylammonium chains.^[^ ^20^ ^]^ Moreover, the interlayer spacing increased at higher temperatures due to the increased rotational disorder. Remarkably, when we mixed 200 mM *Ba* with 200 mM *Oa*, 200 mM *Oa* with 180 mM *DDa*, 180 mM *DDa* with 165 mM *HDa*, and 165 mM *HDa* with 150 mM *ODa* together with an equivalent volume as the antisolvent, from the XRD patterns in Figure S6, Supporting Information, the resulting films were confirmed to be (OA)_2_PbBr_4_, (DDA)_2_PbBr_4_, (HDA)_2_PbBr_4_, and (ODA)_2_PbBr_4_, respectively, indicating that the inorganic slabs only appear to bond with (or at least the layer spacing is only determined by) the longer alkyl chain amines, which is in distinct contrast with the findings of a previous report,^[^ ^21^ ^]^ where the layer spacing of a film prepared using a mixture of two alkylamines adopted a value in between that of the two films prepared using the pure alkylamines.

To confirm the effect of aliphatic alkylamines R = NH_2_(CH_2_)_*m*‐1_CH_3_ with different numbers of carbon atoms *m* on the formation of layered perovskite structures, a model of the transformation process for aliphatic alkylamines R slicing *n* = 3 phase (MA)_4_Pb_3_Br_10_ perovskite into *n* = 1 (RH)_2_PbBr_4_ layered structure was constructed, as shown in Figure 3c. The reaction of formation of *n* = 1 (RH)_2_PbBr_4_ is defined as follows:

(1) $$\begin{array}{c} {\left(\text{MA}\right)}_{4}{\text{Pb}}_{3}{\text{Br}}_{10}+6R+2\text{MABr}=3{\left(\text{RH}\right)}_{2}{\text{PbBr}}_{4}+6Ma \end{array}$$

where aliphatic alkylamines R with different carbon atoms *m* are *Ba* (*m* = 4), *Oa* (*m* = 8), *DDa* (*m* = 12), *HDa* (*m* = 16), and *ODa* (*m* = 18). Figure 3d shows the calculated formation energies of *n* = 1 (RH)_2_PbBr_4_ perovskites, which decrease with the increasing number *m* of carbon atoms in aliphatic alkylamines R. The formation energies of *n* = 1 (BA)_2_PbBr_4_, (OA)_2_PbBr_4_, (DDA)_2_PbBr_4_, (HDA)_2_PbBr_4_, and (ODA)_2_PbBr_4_ are −1.811, −3.057, −4.598, −4.923, and −6.354 eV, respectively. This indicates that the alkylamines with longer chain length are more favorable to slice the 3D perovskite frameworks into the layered 2D structures. This explains why only the *n* = 1 layered perovskite could be yielded as the number *m* of carbon atoms in the alkylamines increased. The combination of longer‐chain alkylamines and the inorganic octahedron generates lower structural energy, and the interaction between them becomes stronger as number *m* of carbon atoms in the alkylamines increases.

It is worth noting that when small amounts of aliphatic alkylamines were applied, the formed hybrid 2D/3D perovskite films exhibited intense green luminescence. The absolute PL quantum yield (PL QY) of perovskite films treated with 100 μL of *Ba* (20 mM), *Oa* (12 mM), *DDa* (65 mM), *HDa* (66 mM), and *ODa* (60 mM) reached 6%, 7%, 38%, 21%, and 18%, respectively. Specific concentrations of the alkylamines in toluene used to produce different films are based on the optimization of their PL QY. We notice that the PL QY for MAPbBr_3_ film was not accurately measurable, due to its low emission efficiency. Absorption and PL spectra of *DDa* (65 mM), *HDa* (66 mM), and *ODa* (60 mM) films are shown in Figure S7, Supporting Information. When *HDa* (66 mM) and *ODa* (60 mM) were used, only the *n* = 1 2D perovskite could be obtained, and an inefficient energy transfer process from this perovskite phase to the 3D perovskite resulted in lower PL QY values for the films treated with *HDa* and *ODa* as compared to the one treated with *DDa*. Although all three films showed strong excitonic peaks at shorter wavelengths (Figure S7a, Supporting Information), they exhibited only one dominant PL peak (Figure S7b, Supporting Information). The SEM image of the MAPbBr_3_/DDa65 film with the highest PL QY is shown in Figure S8, Supporting Information, demonstrating its compact surface and small crystal grains.

**Figure 4:**
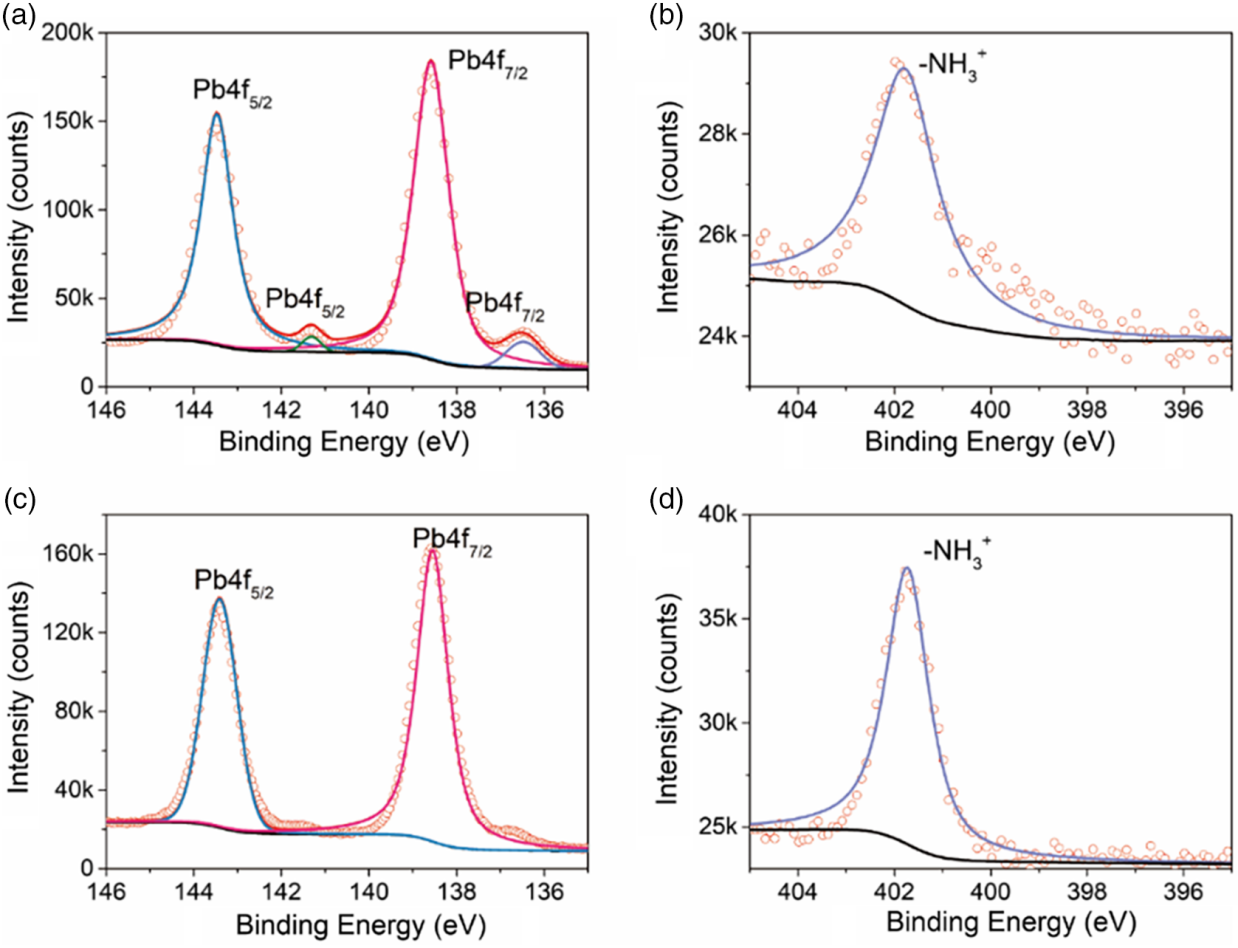
a) Pb4f and b) N1s XPS spectra of the bulk MAPbBr_3_ film, and c) Pb4f and d) N1s XPS spectra of the MAPbBr_3_/Ba200 film. Background curves are shown in black, while those for elemental signals are colored.

We used X‐ray photoelectron spectroscopy (XPS) to study the MAPbBr_3_ and MAPbBr_3_/Ba200 films. **Figure** **4**a,b shows the XPS data for Pb and N elements in the bare MAPbBr_3_ film, respectively. Two dominant peaks located at 138.6 and 143.5 eV can be assigned to Pb4f_7/2_ and Pb4f_5/2_, respectively, both correspond to the Pb^2+^ ions. In addition, two peaks at 136.5 and 141.3 eV can be observed in Figure 4a, which may indicate the existence of some Pb^0^ metal in the films,^[^ ^22^ ^]^ due to the reported tendency for MAPbBr_3_ films to slowly degrade in the air.^[^ ^23^ ^]^ In Figure 4b, there is a strong peak at 401.8 eV in the N1s XPS spectrum, corresponding to an –NH^3+^ group. The atomic ratio of Br and Pb in the MAPbBr_3_ film derived from XPS data is 2.7, which is close to the stoichiometric ratio of MAPbBr_3_. XPS spectra of Pb4f and N1s elements in the MAPbBr_3_/Ba200 film are depicted in Figure 4c,d, respectively. When the MAPbBr_3_ film was treated with 200 mM *Ba*, the intensity of the XPS peaks at the binding energy of 136.5 and 141.3 eV became smaller, suggesting an enhanced stability for the *n* = 1 phase 2D RP perovskite. As shown in Figure 4d, there is only one sharp peak located at 401.8 eV, which can be assigned to –NH^3+^. Since *Ba* has a rather low boiling point (≈78 °C), which is lower than the heating temperature (85 °C) of the annealing treatment applied to prepare the films, the residual *Ba* left in the film would totally evaporate after the annealing process. Thus, the existence of –NH^3+^ implies that *Ba* was protonated to butylammonium cation (BA^+^) during the spin‐coating. The atomic ratio of Br and Pb in the MAPbBr_3_ film treated with 200 mM *Ba* was found to be 3.7, which further confirms the formation of the (BA)_2_PbBr_4_ perovskite. Pb4f and N1s XPS spectra of the MAPbBr_3_ films treated with 120 mM *Oa*, 165 mM *HDa*, and 150 mM *ODa* are shown in Figure S9, Supporting Information. It is noteworthy that the XPS peaks of Pb^0^ metal were absent in the Pb4f XPS spectra of those films, which implies that the 2D perovskites with long‐chain alkylamines have better in‐air stability than the bare MAPbBr_3_ film. Meanwhile, the N1s XPS spectra can be decomposed into two distinct peaks, as shown in Figure S9d and S9f, Supporting Information. The small peak at 399 eV can be assigned to –NH_2_, and the reason for this is that *HDa* and *ODa* have relatively high boiling points (≈332 and ≈232 °C, respectively), and thus they may remain in the perovskite films after heating. Thus, from the abovementioned results, the introduced alkylamines were protonated into ammonium salts, which occupied the A site of the MAPbBr_3_ perovskite, leading to the formation of 2D layered films.

As toluene is a nonpolar solvent and DMSO is a polar aprotic solvent, it is difficult for *Ba* to be protonated in toluene or DMSO. To confirm this, Fourier‐transform infrared spectroscopy (FTIR) was carried out, with the data presented in Figure S10, Supporting Information. For *Ba* dissolved in toluene, FTIR peaks corresponding to the N–H stretching mode (3289 cm^−1^) and to the N–H scissor mode (1600 cm^−1^) are present at the same positions as for the reference pure *Ba* liquid. Similarly, there was no shift of the N–H stretching mode or N–H scissor mode for *Ba* dissolved in DMSO. This confirms that *Ba* could not be protonated in DMSO or toluene solvents. In contrast, in the MAPbBr_3_ perovskite, the MA^+^ cation is a weak acid, whereas the *Ba* is a strong base, as the basicity increases with the increasing length of the alkyl chain. In such cases, proton transfer from MA^+^ to Ba can occur, which would render *Ba* to become a BA^+^ cation. BA^+^ cations interact with the perovskite inorganic frameworks through hydrogen bonds, and subsequently scission anionic perovskite slabs into separate layers, yielding 2D RP perovskite as schematically shown in Figure 2c.

**Figure 5:**
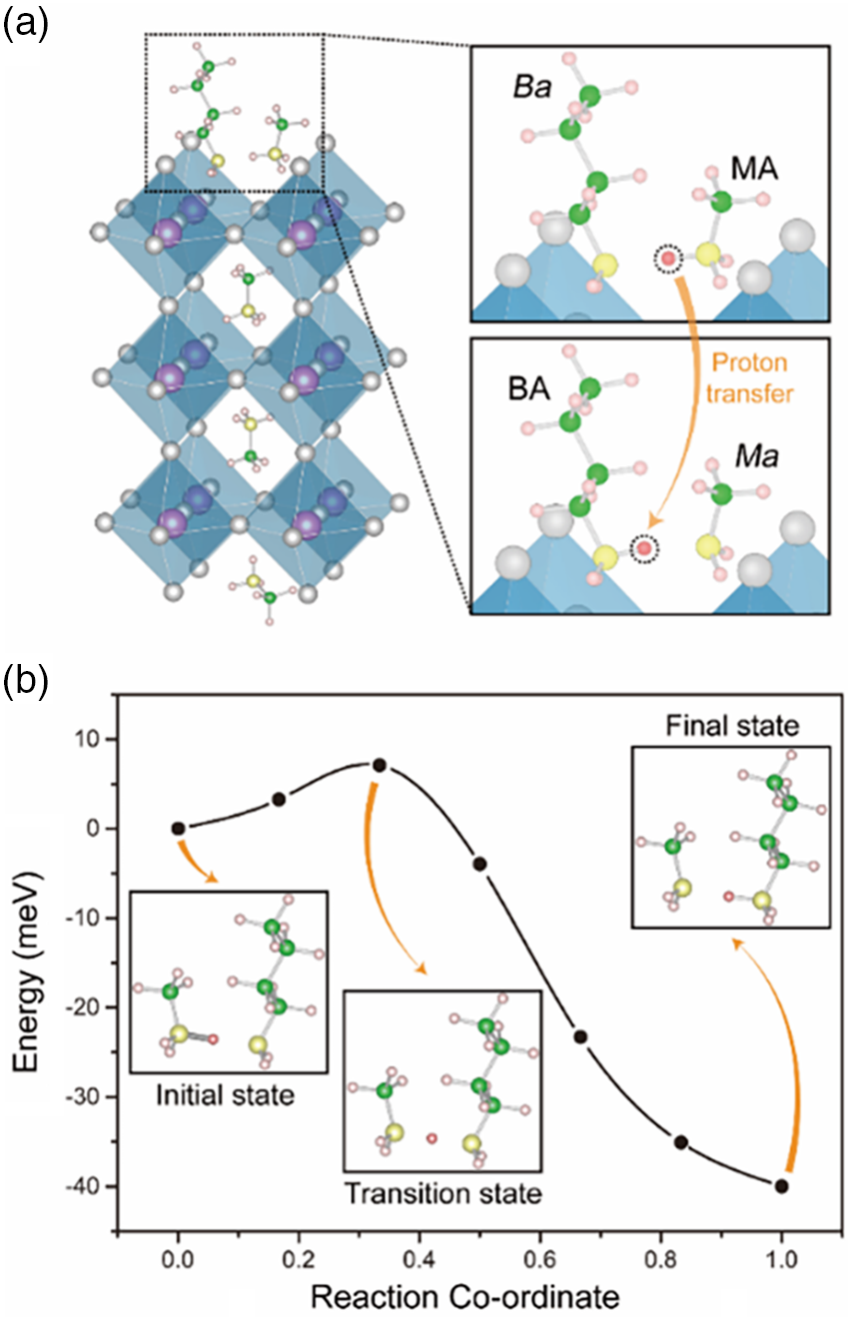
a) Schematic diagram of the proton transfer process between *Ba* and MA^+^ cation (zoomed‐in inside the dashed box) on the surface of *n* = 3 phase (MA)_4_Pb_3_Br_10_ perovskite. The transferred H atoms are marked by the dashed circles in the right panel. At the initial state (upper panel), the H atom is bonded with the N atom on MA. The H atom is transferred from the N atom of MA to the N atom of *Ba* at the final state (lower panel). b) Schematic of NEB images of proton transfer process. A transition state is between the initial state and the final state, whereas the H atom is between the N atoms on MA and *Ba*.

To confirm the occurrence of proton transfer during the slicing of the 3D perovskite into a layered structure by alkylamines, a model was constructed to simulate the process of proton transfer as shown in **Figure** **5**a. Unprotonated *Ba* attacks MA^+^ on the surface of *n* = 3 (MA)_4_Pb_3_Br_10_ perovskite. The right panel in Figure 5a shows the initial (upper panel) and final (lower panel) states of the proton transfer process. The H atom in the dashed circle in the right panel of Figure 5a on MA^+^ is transferred to *Ba* and protonates it. We studied the proton transfer process between the two states by performing the nudged‐elastic‐band (NEB) calculation as shown in Figure 5b. The distances from the transferred H atom to the N atoms on MA^+^ and *Ba* are *d*_1_ and *d*_2_ (listed in Table S1, Supporting Information), respectively. With the transfer of H atoms from MA^+^ to *Ba*, *d*_1_ increases from 1.154 to 1.589 Å and *d*_2_ decreases from 1.518 to 1.123 Å. Values of *d*_1_ and *d*_2_ of the transition state structure at the energy barrier are 1.274 and 1.344 Å, which shows that the H atom is situated between the N atoms on *Ma* and *Ba*. The low energy of the barrier (7.09 meV) means that proton transfer can easily occur, and the increase in temperature can further facilitate the proton transfer. During the proton transfer process, there are three states: the initial state, the transition state, and the final state. In the initial state, the H atom is bonded with the N atom at the MA^+^ cation. In the transition state, MA^+^ loses the H atom, which then falls between the methylamine and the introduced aliphatic alkylamines. In the final state, the H atom is bonded with the N atom at the introduced aliphatic alkylamines, forming the 2D layered perovskites. Between the transition state and the initial state, there is a small energy barrier. For short alkyl chain amines, this energy barrier can be overcome at room temperature, while for long alkyl chain amines, an elevated annealing temperature is needed for proton transfer to become complete.

In Figure S11, Supporting Information, we demonstrate the possibility to tune the halide content in the *n* = 1 (ODA)_2_Pb_2_X_4_ (X = Br, I) films. The absorption energies spanning from the UV to the green can be well controlled by modifying their mixed halide stoichiometry. The absorption spectra of other perovskites, namely tin‐based MASnBr_3_ and all‐inorganic CsPbBr_3_ perovskites, which were made by applying an identical fabrication procedure, are shown in Figure S12, Supporting Information. The excitonic peak of the MASnBr_3_ absorption spectrum at 540 nm shows a significant blueshift when different alkylamines were utilized (Figure S12a, Supporting Information), indicating the formation of an analogous 2D Sn‐based perovskite. However, when the A‐site cation of the perovskite was changed from MA^+^ into Cs^+^, no blueshift of the absorption peaks was observed (Figure 2a versus Figure S12b, Supporting Information). This provides another hint on the importance of the proton transfer in the process because alkylamines could not be protonated by the Cs^+^ cation through proton transfer.

We have further explored the effects of the annealing temperature on MAPbBr_3_ films made with different alkylamines. As shown in Figure S13, Supporting Information, for MAPbBr_3_ treated with *Ba*, *Oa*, and *DDa*, the absorption spectra showed negligible changes at different annealing temperatures. However, for MAPbBr_3_ treated with long‐chain alkylamines, such as *HDa* and *ODa*, the intensity of the excitonic absorption peaks corresponding to their 2D layered perovskites first increased with rising temperature from 25 to 65 °C, and then remained almost unchanged when the annealing temperature reached 65°. On the contrary, the intensity of the absorption peak at 518 nm corresponding to MAPbBr_3_ gradually decreased with increased annealing temperature (an annealing time of 10 min was used throughout). This observation indicates that the proton transfer process needs to overcome much larger energy barriers for the *HDa* and *ODa* cases, and thus the elevated annealing temperature facilitated proton transfer, which is consistent with the calculation results (Figure 5b). XRD patterns of the MAPbBr_3_/ODa150 films, without annealing and annealing at 105 °C for 10 min, are shown in Figure S14, Supporting Information. The non‐annealed perovskite film exhibited poor crystallinity, and there was a broad peak at 15° indicating the presence of the 3D perovskite phase. In contrast, when the film was annealed at 105 °C, this diffraction peak vanished, giving way to well pronounced, periodic diffraction peaks which correspond to *n* = 1 2D MAPbBr_3_ perovskite. We further measured the PL spectra of the MAPbBr_3_/DDa90 film from the front (perovskite side) and back (glass side) of the substrate. As shown in Figure S15, Supporting Information, the two PL spectra are almost identical, indicating that the *n* = 1 2D perovskite phase is homogeneously distributed along the thickness of the film.

## 3 Conclusions

In summary, we have systematically studied interactions between aliphatic alkylamines and different metal halide perovskites. Our experimental and theoretical results show that the aliphatic alkylamines can be easily protonated by the MA^+^ cation in perovskite. Longer‐chain alkylamines are more prone to bond with the lead halide frameworks underpinning the perovskite phases. In the MAPbBr_3_ case, by adjusting the alkylamine concentration, the 3D/2D hybrid perovskite and eventually a pure *n* = 1 phase perovskite can be obtained. Furthermore, only *n* = 1 phase perovskite could be generated when longer‐chain alkylamines (carbon atoms equal to 12 or greater) are introduced into the MAPbBr_3_. Our study offers new insights into the fabrication of highly luminescent (PL QY up to 38%) 3D/2D hybrid perovskite films and highly oriented *n* = 1 phase 2D perovskites, which are potentially useful for optoelectronic applications.

## 4 Experimental Section

4.1

4.1.1

##### Chemicals

Butylamine (*Ba*, 99.5%), dodecylamine (*DDa*, 99%), hexadecylamine (*HDa*, 98%), octadecylamine (*ODa*, 99%), and toluene (99.8%) were purchased from Sigma‐Aldrich. Octylamine (*Oa*, 99 %) was purchased from Acros Organic. DMSO (anhydrous, 99.9%), DMF (anhydrous, 99.9%), methylammonium bromide (MABr, 99.5%), lead bromide (PbBr_2_, 99.9%), methylammonium iodide (MAI, 99.5%), lead iodide (PbI_2_, 99.9%), and tin bromide (SnBr_2_, 99.99%) were purchased from Advanced Election Technology Co., Ltd. All reagents were used as received without further purification.

##### Preparation of Perovskite Precursor and Antisolvent Solutions

The MAPbX_3_ (X = Br, I) solution was prepared by adding 1 mmol PbX_2_ and 1 mmol MAX in 1 mL DSMO, followed by vigorously stirring for 12 h in a glove box. The MASnBr_3_ precursor solution was prepared by dissolving 1 mmol SnBr_2_ and 1 mmol MABr in 1 mL DMSO with vigorously stirring for 12 h in a glove box. The *Ba* containing antisolvents were prepared by dissolving 2, 5, 10, and 20 μL *Ba* in 1 mL toluene, respectively, with final concentrations of 20, 50, 100, and 200 mM. The *Oa* containing antisolvents were prepared by dissolving 2, 5, 10, and 20 μL *Oa* in 1 mL toluene, respectively, with final concentrations of 12, 30, 60, and 120 mM. The *DDa* containing antisolvents were prepared by dissolving 5, 10, 20, and 40 μL *DDa* which was preheated at 60 °C for 30 min in 1 mL toluene, respectively, with final concentrations of 22, 45, 90, and 180 mM. The *HDa* containing antisolvents were prepared by dissolving 0.024, 0.036, 0.072, and 0.12 g *HDa* in 3 mL toluene, respectively, with concentrations of 33, 50, 100, and 165 mM. After vigorous stirring for 24 h in the glove box, the *HDa* antisolvents were filtered through polytetrafluoroethylene (PTFE) filters with a 20 nm pore size. The *ODa* containing antisolvents were prepared by dissolving 0.024, 0.036, 0.06, and 0.12 g *ODa* in 3 mL toluene, respectively, with concentrations of 30, 45, 75, and 150 mM. After vigorous stirring for 24 h in the glove box, the *ODa* antisolvents were filtered through PTFE filters.

##### Fabrication of Perovskite Films

Silica substrates were sequentially cleaned in acetone, ethanol, and deionized water by ultrasonication for 10 min. After drying the cleaned substrates with nitrogen gas, the substrates were treated with ozone for 15 min to obtain a hydrophilic surface. Subsequently, 50 μL of the perovskite precursor was deposited on the glass substrate and spin‐coated at 5000 rpm for 60 s. At the moment when the color of the films changed to pale yellow, 100 μL of antisolvent was added onto the still spinning film. Then, the perovskite films were annealed at 85 °C for 10 min. The entire spin‐coating and annealing processes were conducted in a glove box.

##### Calculation Details

First‐principles density functional theory calculations were performed by using the plane wave projection augmented wave pseudopotential method, which is implemented in the code of the Vienna Ab‐initio Simulation Package.^[^ ^24^ ^]^ The generalized gradient approximation formulated by Perdew, Burke, and Ernzerhof was used for the exchange and correlation function.^[^ ^25^ ^]^ To simulate the layered perovskite structures, the vacuum space perpendicular to the direction of the perovskite layer was set over 25 Å to avoid the interaction between adjacent layers. The kinetic energy cutoff of the plane wave basis set is set to 500 eV. In the electronic Brillouin zone integration, we use a k‐point grid with a grid spacing of 2π × 0.08 Å^−1^ or less. The tolerance for electron minimization is 10^−4^ eV, and the force tolerance for ion relaxation is 0.05 eV Å^−1^. The energy barrier of the proton transfer process was calculated using the NEB method combined with the climbing image method.^[^ ^26^ ^]^ Considering that van der Waals interaction is very important for organic–inorganic hybrid perovskite, optB86b‐vdW functional compound was used.^[^ ^27^ ^]^

##### Characterization

SEM images were obtained on a scanning electron microscope (Quanta 450 FEG) with an operating voltage of 10 kV. Absorption spectra were measured on an ultraviolet−visible spectrophotometer (Cary 50, Varian). XRD measurements were carried out on a Philips X‐Pert X‐ray diffractometer (Cu Kα radiation, *λ* = 1.5418 Å). AFM images were collected on a Vesco 3100 multimode V AFM instrument. Steady‐state PL spectra of the prepared films were measured at room temperature by using a fluorescence spectrophotometer (Edinburgh Instruments FLS 920). PL emissions were measured under 320 nm UV light excitation provided by a xenon lamp. Elemental compositions of perovskite films were obtained on an XPS instrument (Thermo Fisher Scientific K‐Alpha) with an Al Kα radiation source. FTIR spectra were collected on an FT‐IR spectrophotometer (Perkin Elmer 16 PC).

## Conflict of Interest

The authors declare no conflict of interest.

## Supporting information

Supplementary Material

## Data Availability

The data that support the findings of this study are available in the supplementary material of this article.
