# Supplementary material for: Proton Transfer‐Driven Modification of 3D Hybrid Perovskites to Form Oriented 2D Ruddlesden–Popper Phases
Source: Small Sci. 2021 Dec 23;2(3):2100114. doi: 10.1002/smsc.202100114 (PMC11935931; doi:10.1002/smsc.202100114)
Supplement: Supplementary file 1 — Supplementary Material [file SMSC-2-2100114-s001.pdf]

Supporting Information

Proton Transfer Driven Modification of Three-Dimensional Hybrid Perovskites to form Oriented Two-Dimensional Ruddlesden-Popper Phases

Zonghui Duan[1], Guangren Na[2], Shixun Wang[1], Jiajia Ning[1], Bangyu Xing[2], Fei Huang[3], Arsenii S. Portniagin[1], Stephen V. Kershaw[1], Lijun Zhang[2,*] and Andrey L. Rogach[1,*]

[1]Department of Materials Science and Engineering, and Centre for Functional Photonics (CFP), City University of Hong Kong, Hong Kong SAR 999077, P. R. China

[2]State Key Laboratory of Superhard Materials, Key Laboratory of Automobile Materials of MOE, College of Materials Science and Engineering, Jilin University, Changchun 130012, P. R. China

[3]Institute for Advanced Materials and Technology, University of Science and Technology Beijing, Beijing 100083, P. R. China

These authors contributed equally: Zonghui Duan, Guangren Na

Corresponding authors: lijun_zhang@jlu.edu.cn (LZ), andrey.rogach@cityu.edu.hk (ALR)

In the Figure captions, the following abbreviations are used for denoting aliphatic alkylamines with varying aliphatic chain length: butylamine (*Ba*, 4 carbon atoms), octylamine (*Oa*, 8 carbon atoms), dodecylamine (*DDa*, 12 carbon atoms), hexadecylamine (*HDa*, 16 carbon atoms), and octadecylamine (*ODa*, 18 carbon atoms).

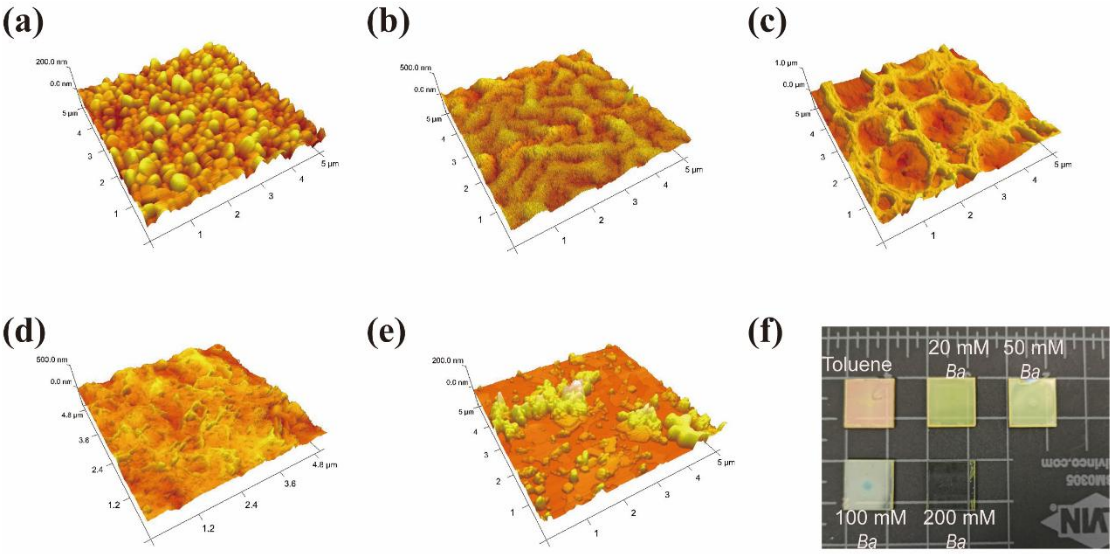

Figure S1. AFM images of a) pristine $MAPbBr_3$ film and $MAPbBr_3$ films obtained upon treatment with b) 20 mM *Ba*, c) 50 mM *Ba*, d) 100 mM *Ba* and e) 200 mM *Ba*. f) Photograph taken under day light, showing different colors of a pristine $MAPbBr_3$ film (“toluene”), and $MAPbBr_3$ films obtained with 20 mM *Ba*, 50 mM *Ba*, 100 mM *Ba* and 200 mM *Ba*, as labelled on the image.

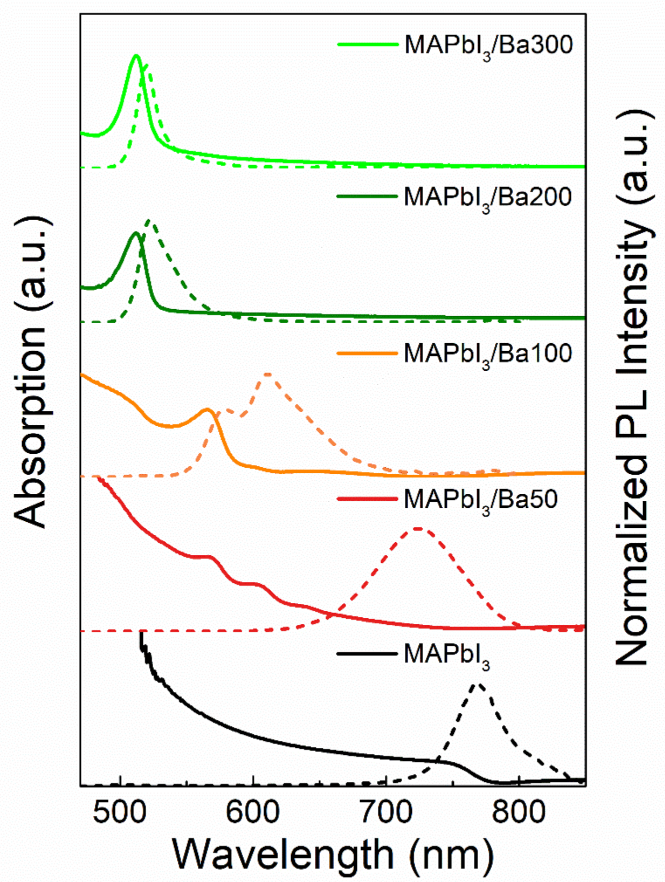

Figure S2. Absorption (solid lines) and PL (dashed lines) spectra of $MAPbI_3$ films fabricated using pure toluene, 50 mM *Ba*, 10 mM *Ba*, 200 mM *Ba*, and 300 mM *Ba* as antisolvents.

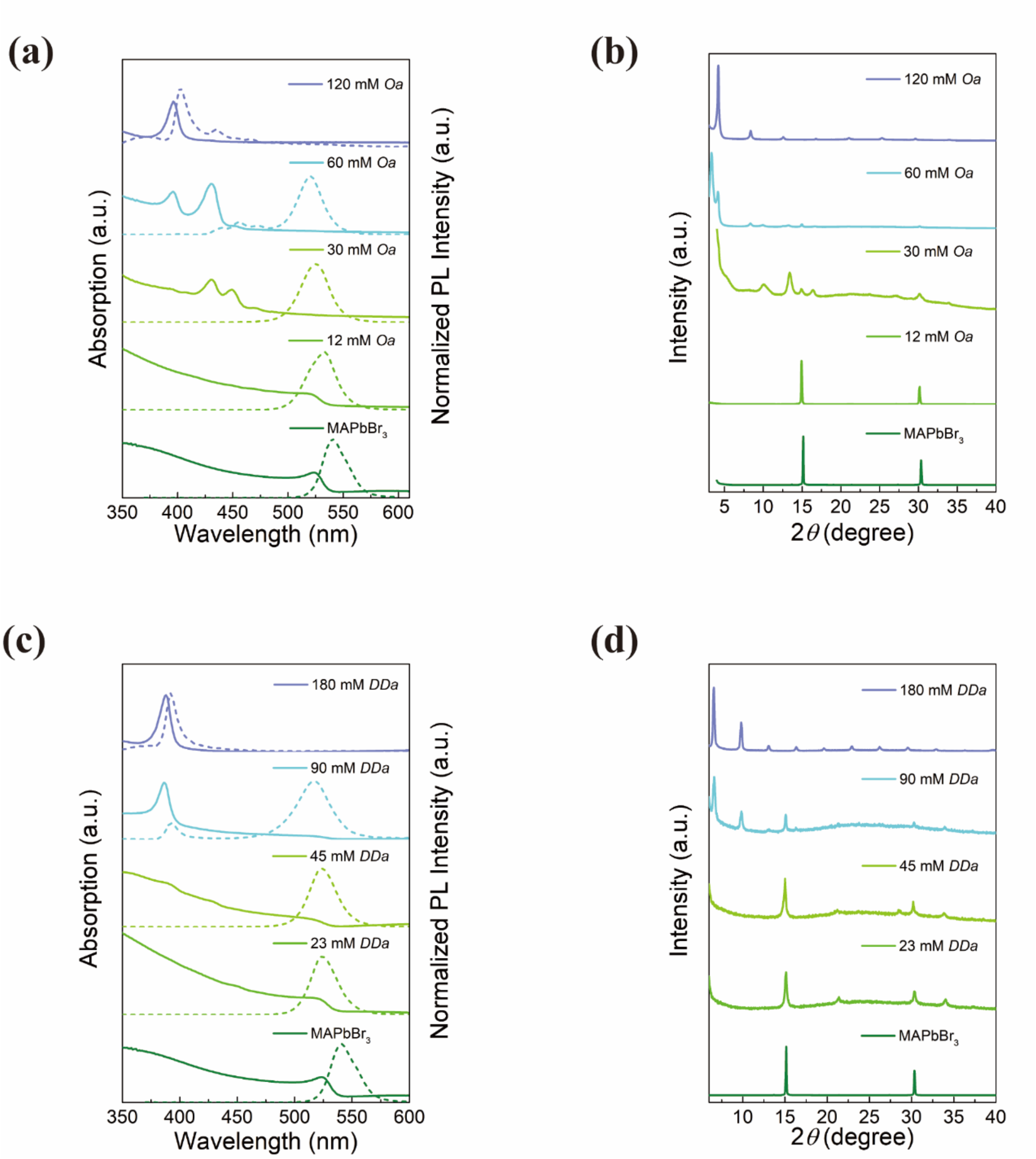

Figure S3. a) Absorption (solid lines) and PL (dashed lines) spectra, and b) XRD patterns of $MAPbBr_3$ films fabricated with pure toluene, 12 mM *Oa*, 30 mM *Oa*, 60 mM *Oa*, and 120 mM *Oa* as antisolvents. c) Absorption (solid lines) and PL (dashed lines) spectra, and d) XRD patterns of $MAPbBr_3$ films fabricated with pure toluene, 22 mM *DDa*, 45 mM *DDa*, 90 mM *DDa*, and 180 mM *DDa* as antisolvents.

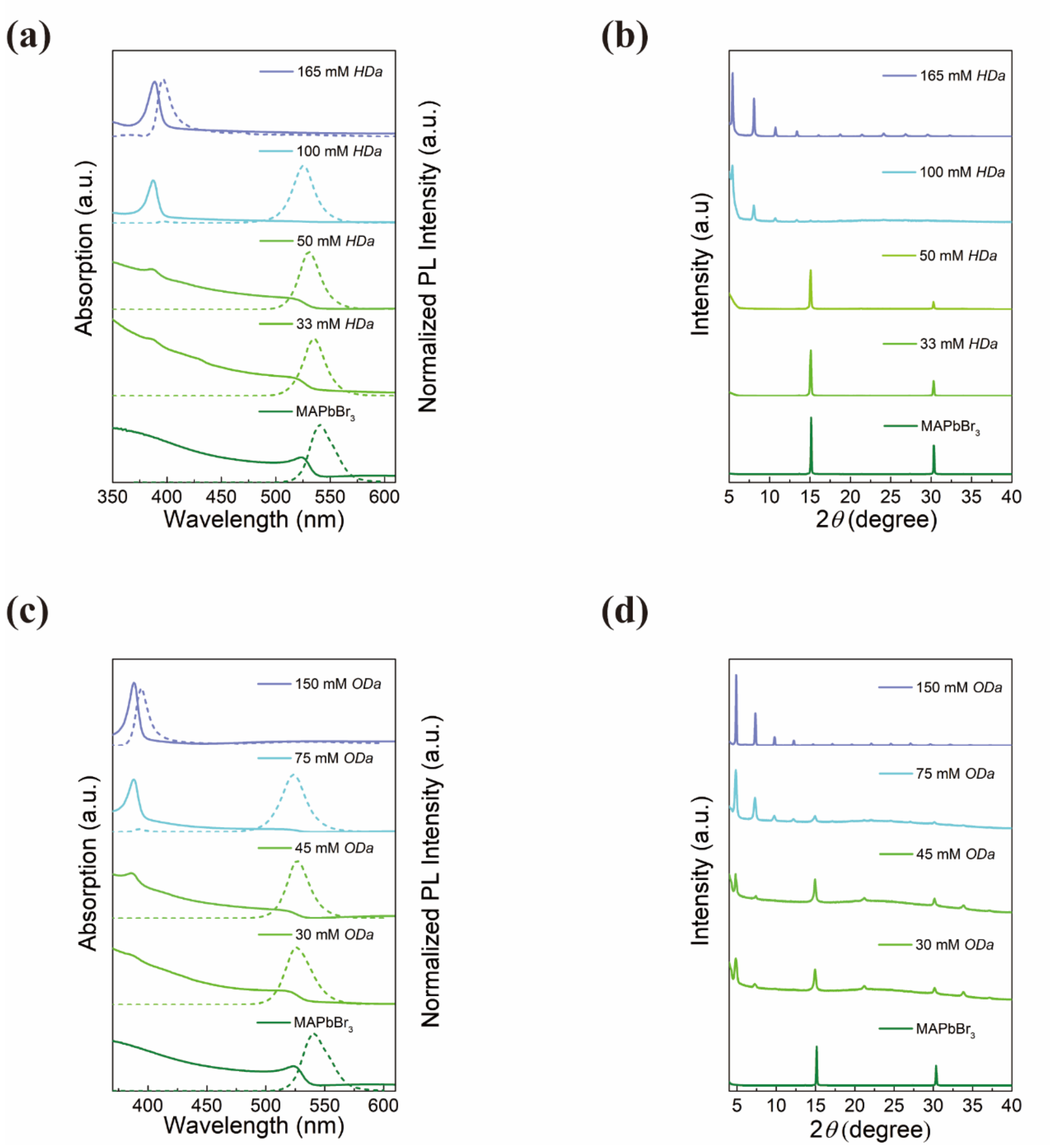

Figure S4. a) Absorption (solid lines) and PL (dashed lines) spectra, and b) XRD patterns of $MAPbBr_3$ films fabricated with pure toluene, 33 mM *HDa*, 50 mM *HDa*, 100 mM *HDa*, and 165 mM *HDa* as antisolvents. c) Absorption (solid lines) and PL (dashed lines) spectra, and d) XRD patterns of $MAPbBr_3$ films fabricated with pure toluene, 30 mM *ODa*, 45 mM *ODa*, 75 mM *ODa*, and 150 mM *ODa* as antisolvents.

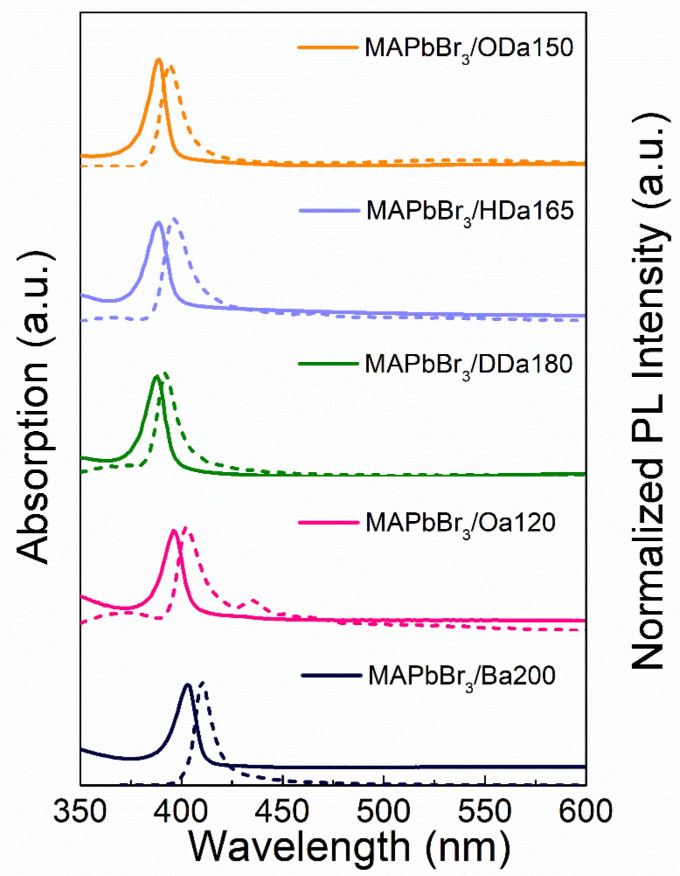

Figure S5. Absorption (solid lines) and PL (dashed lines) spectra of n = 1 phase 2D perovskite films made with different ammonium cations, as indicated on the frame.

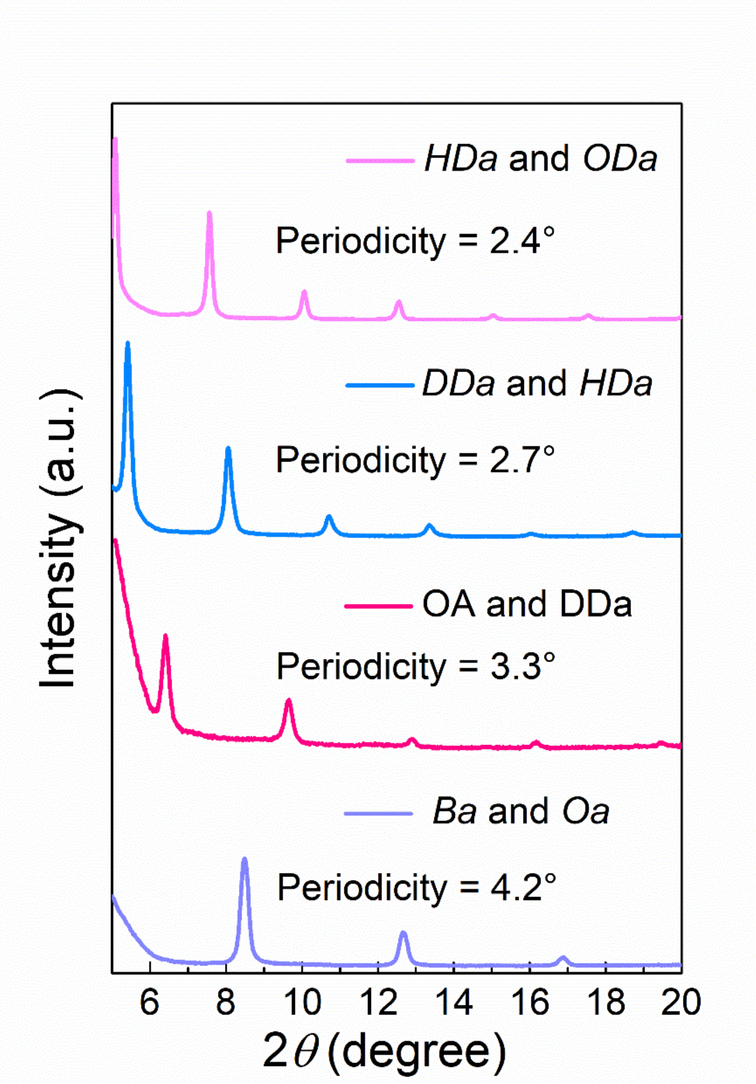

Figure S6. XRD patterns of $MAPbBr_3$ films made with equivalent volume mixtures of 200 mM *Ba* and 120 mM *Oa*, 120 mM *Oa* and 180 mM *DDa*, 180 mM *DDa* and 165 mM *HDa*, and 165 mM *HDa* and 150 mM *ODa*.

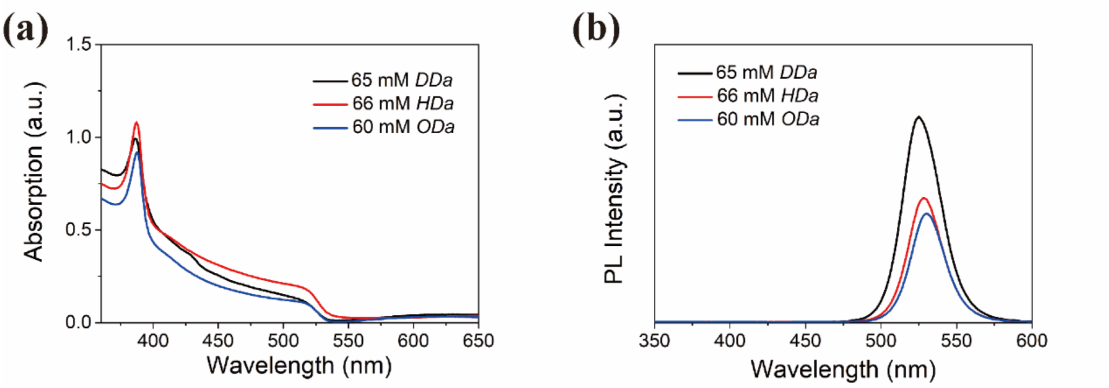

Figure S7. (a) Absorption (a) and (b) PL spectra of $MAPbBr_3$ films fabricated with 65 mM *DDa*, 66 mM *HDa* and 60 mM *ODa*.

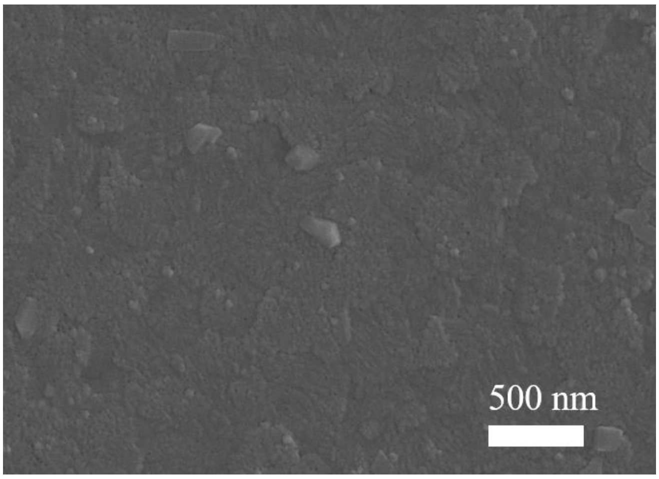

Figure S8. SEM image of $MAPbBr_3$ films fabricated with 65 mM *DDa*.

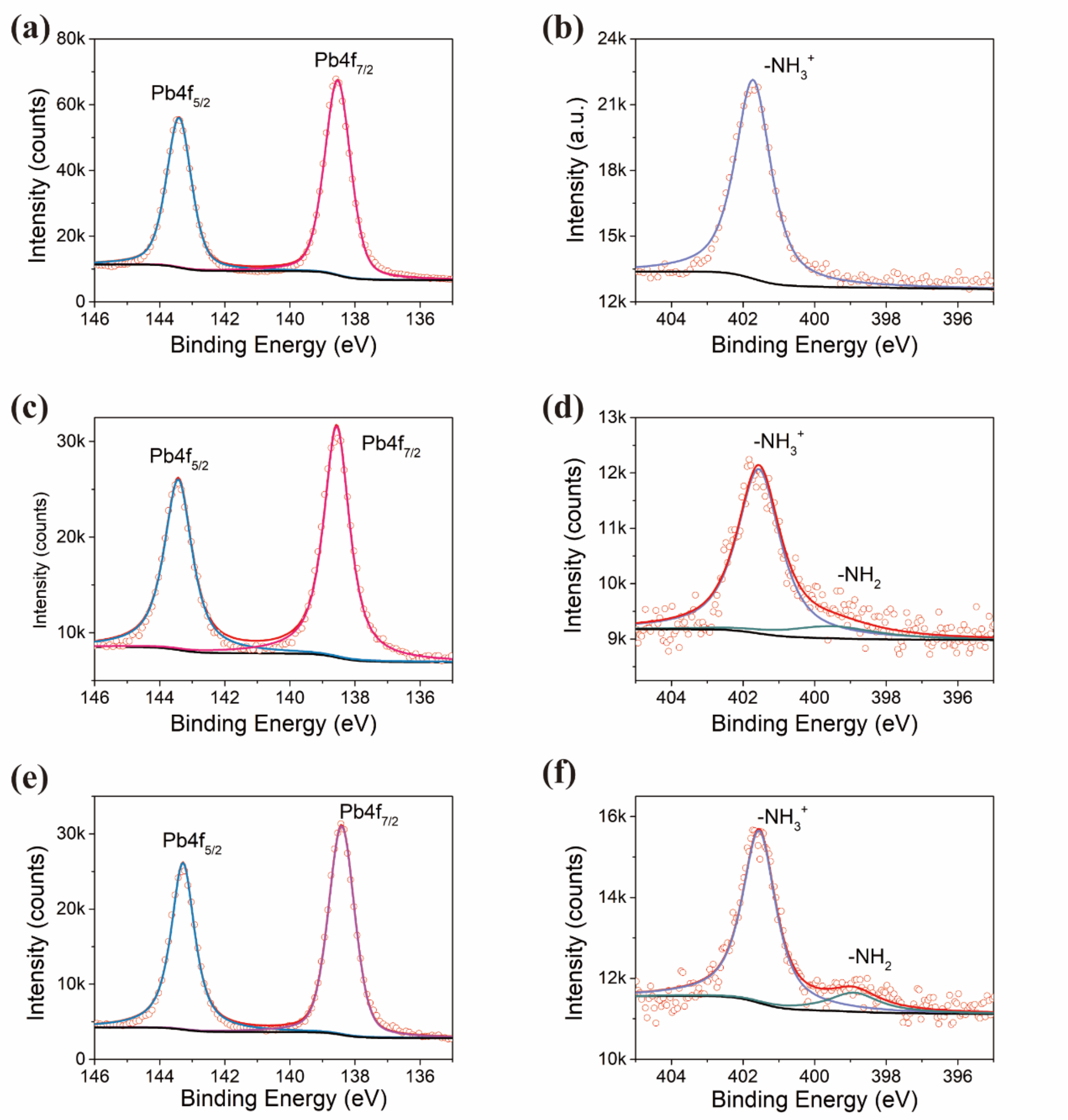

Figure S9. a) Pb4f and b) N1s XPS spectra of $MAPbBr_3$/Oa120 film. c) Pb4f and d) N1s XPS spectra of $MAPbBr_3$/HDa165 film. e) Pb4f and f) N1s XPS spectra of $MAPbBr_3$/ODa150 film.

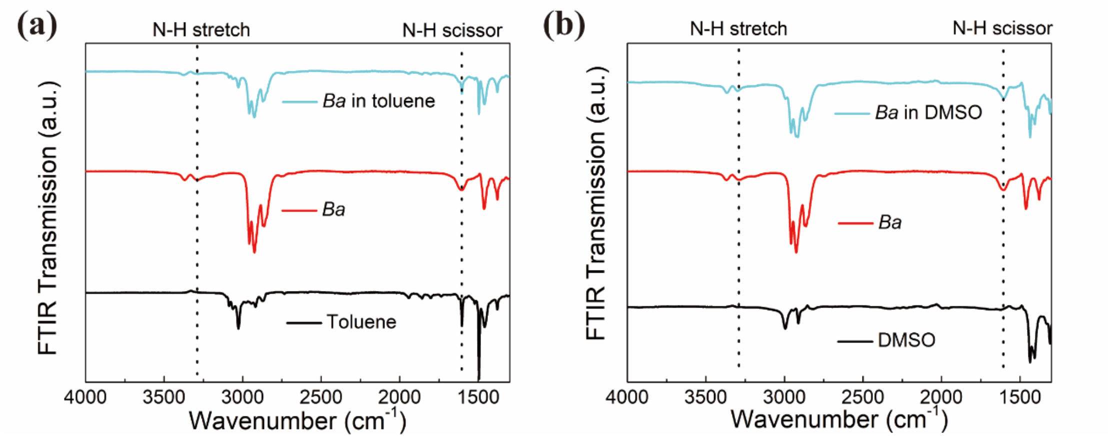

Figure S10. a) FTIR spectra of *Ba*, toluene, DMSO, *Ba* dissolved in toluene and b) *Ba* dissolved in DMSO. The two dash lines in the panels show the position of N-H stretch mode and N-H scissor mode, respectively.

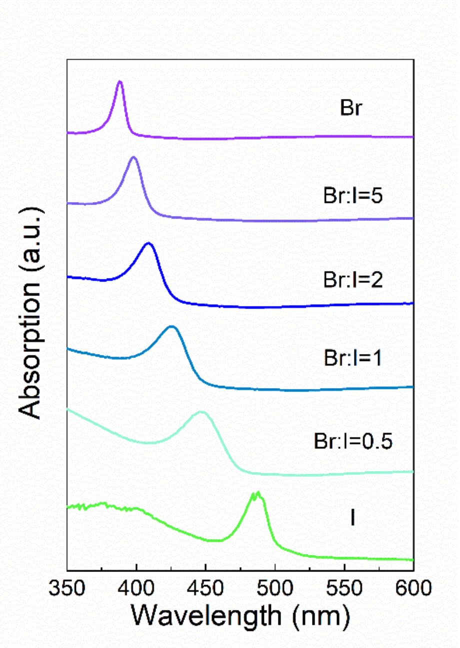

Figure S11. Absorption spectra of n = 1 phase $(ODA)_2Pb_2X_4$ perovskite films showing continuous tunability by tuning the halide (Br/I ratio) composition, as indicated on the frame.

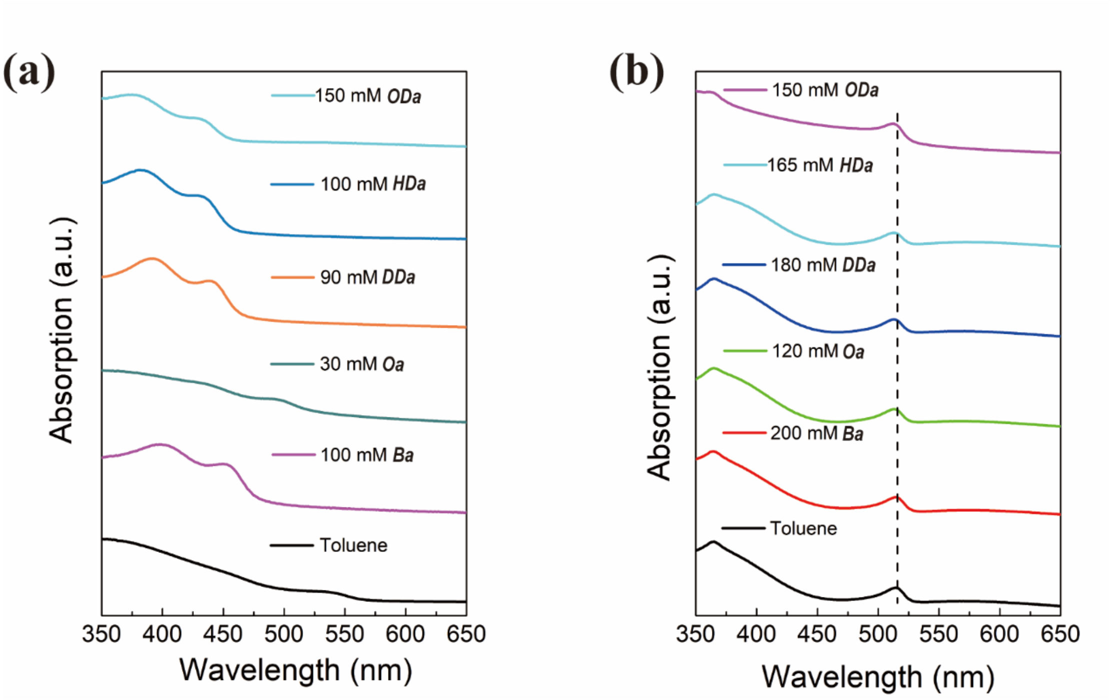

Figure S12. Absorption spectra of a) $MASnBr_3$ and b) $CsPbBr_3$ perovskite films fabricated with different alkylamines. The annealing temperature was 85 ℃ in all cases.

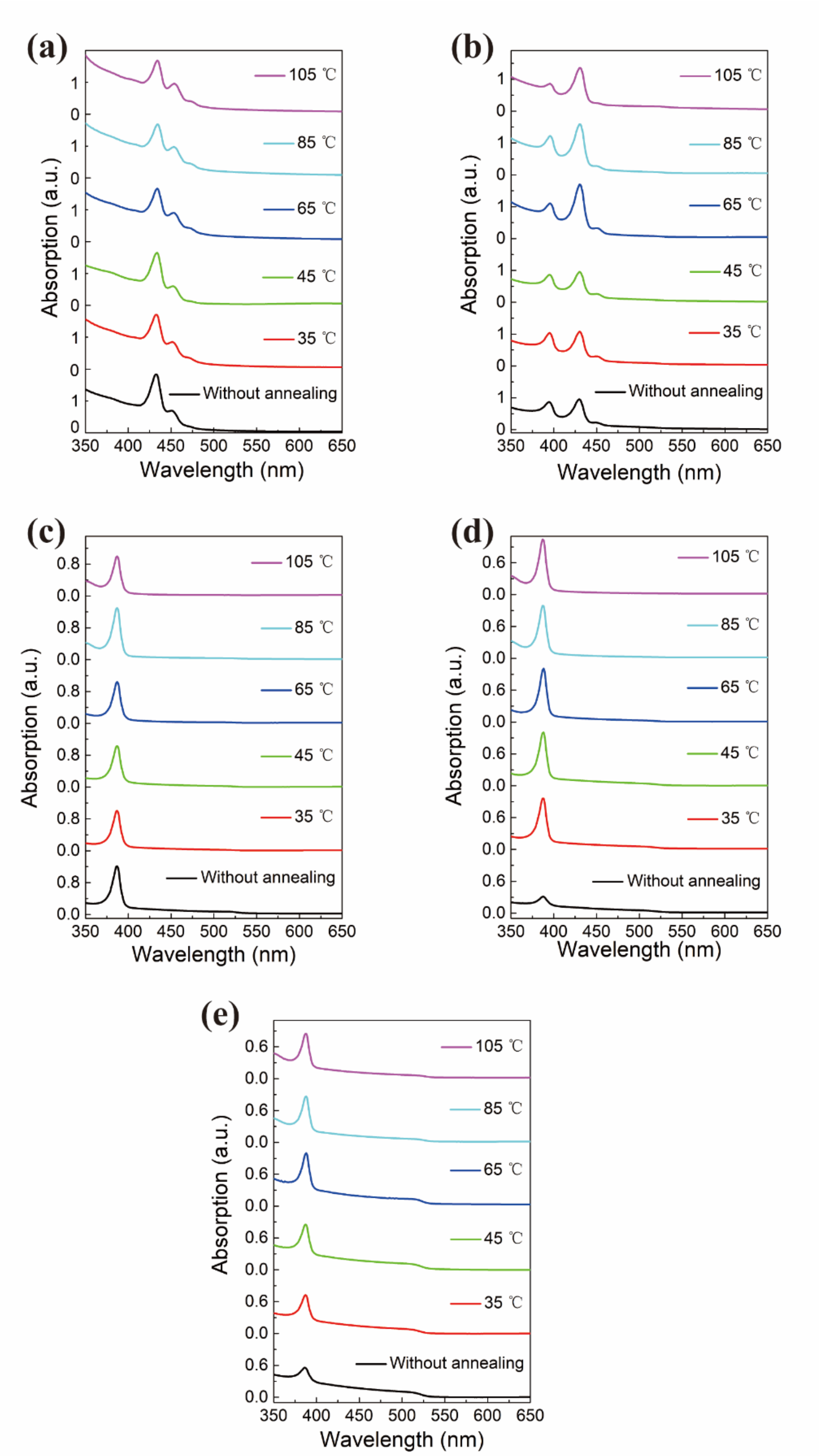

Figure S13. Absorption spectra of $MAPbBr_3$ films fabricated with a) 100 mM *Ba*, b) 60 mM *Oa*, c) 135 mM *DDa*, d) 165 mM *HDa* and e) 150 mM *ODa* at different annealing temperatures, as indicated on the frames. The heating time was fixed at 10 min for all cases.

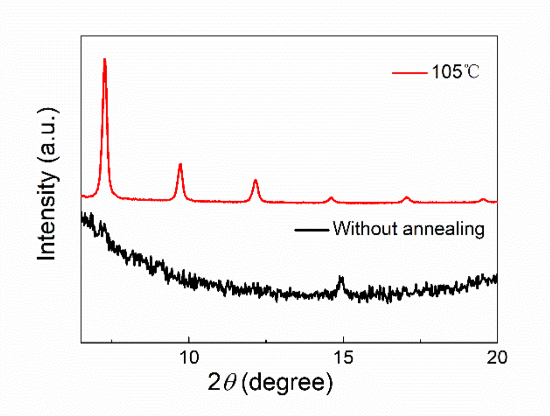

Figure S14. XRD patterns of $MAPbBr_3$ films fabricated with 150 mM *ODa*, without annealing (black line) and annealed at 105°C (red line).

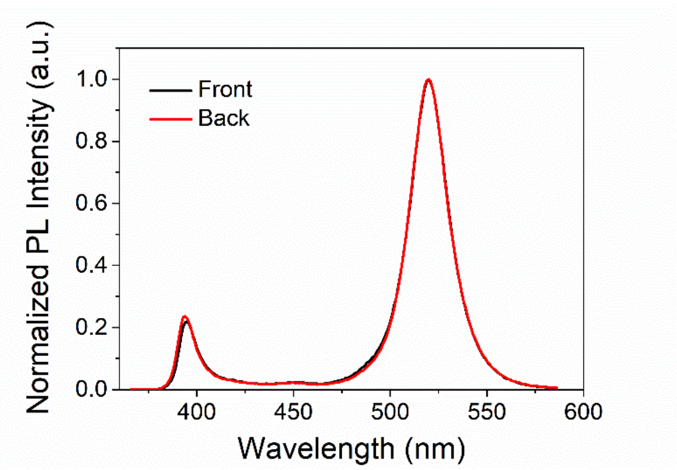

Figure S15. Normalized PL spectra of the $MAPbBr_3$/DDa90 film measured from the front (perovskite side) and back (glass side) of the substrate.

Table S1. The distances from the transferred H atom to the N atoms on MA (d_1) and *Ba* (d_2) cations of all the structures relaxed by nudged-elastic-band (NEB) approach in Figure 5b in the main manuscript. The structures transitioning from the initial state to the final state are marked as S1 to S7 according to changing reaction coordinate.

	S_1	S_2	S_3	S_4	S_5	S_6	S_7
d_1 (Å)	1.154	1.193	1.274	1.376	1.475	1.544	1.589
d_2 (Å)	1.518	1.448	1.344	1.246	1.173	1.139	1.123
